# Supplementary material for: Structure, Functionality, Compatibility with Pesticides and Beneficial Microbes, and Potential Applications of a New Delivery System Based on Ink-Jet Technology
Source: Sensors (Basel). 2023 Mar 12;23(6):3053. doi: 10.3390/s23063053 (PMC10058129; doi:10.3390/s23063053)
Supplement: Supplementary file 1 [file sensors-23-03053-s001.zip › sensors-2231258-supplementary.pdf]

Figure S1. Possible configuration of drop on demand thermal inkjet printheads.

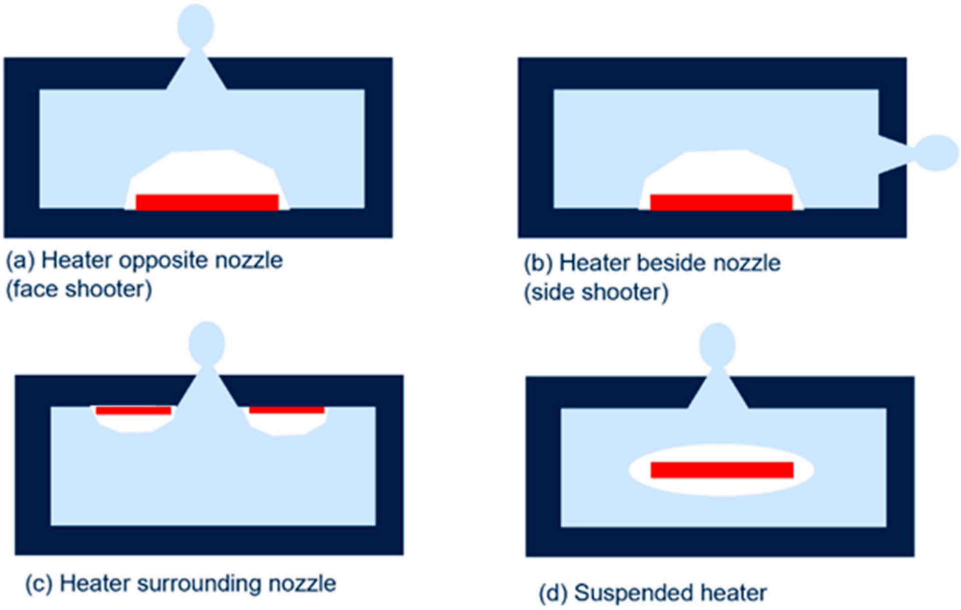

Figure S2. Representative images of the tests with abamectin and deltamethrin with *G. mellonella* and *S. carnacia*. The images show the 3.3 cm diameter Petri dishes hosting the *G. mellonella* larvae and the multi-well plate hosting the *S. carnaria* larvae.

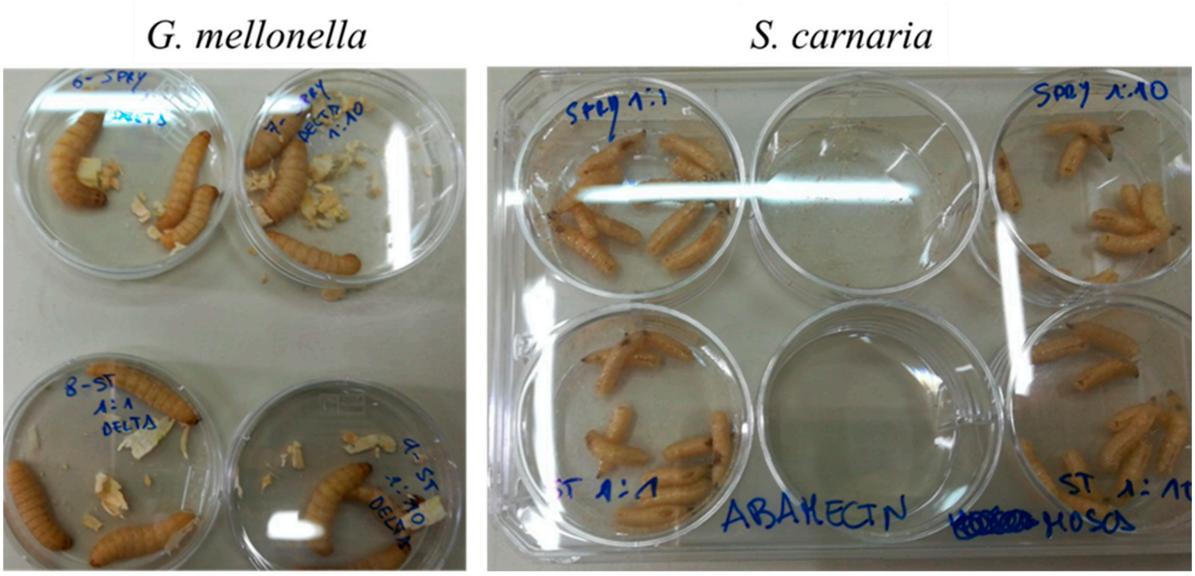

Figure S3. Image of the DEMO developed and used during experiments with beneficial microorganisms applied to different crops and cultivation systems. A: *Lactuca sativa* in pots; B: *Eruca vesicaria* in pot; C: *Eruca vesicaria* in squared petri dish.

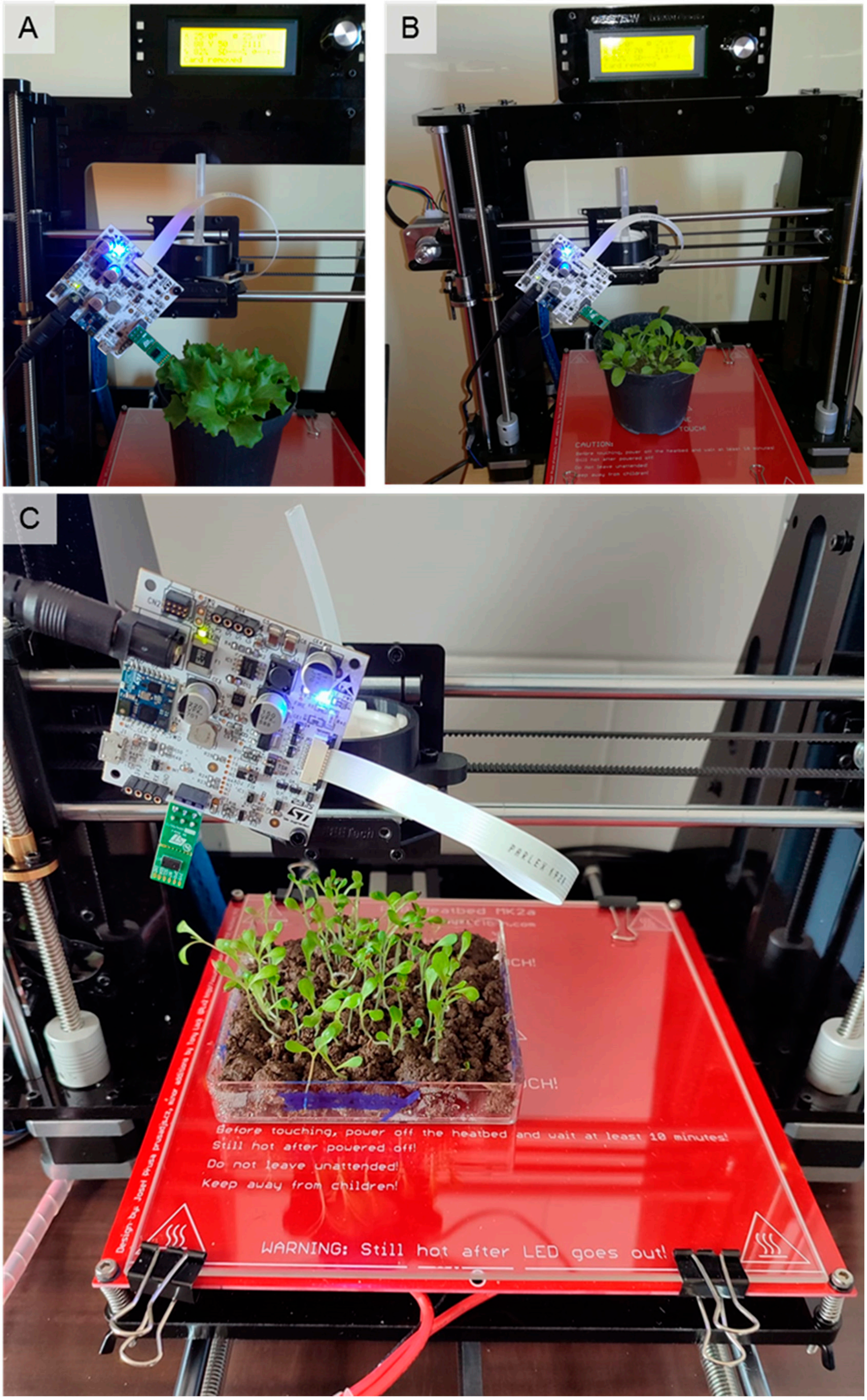

Figure S4. A, B. Examples of "paths" planned for the use of the DEMO applied to the cultivation of microgreens.

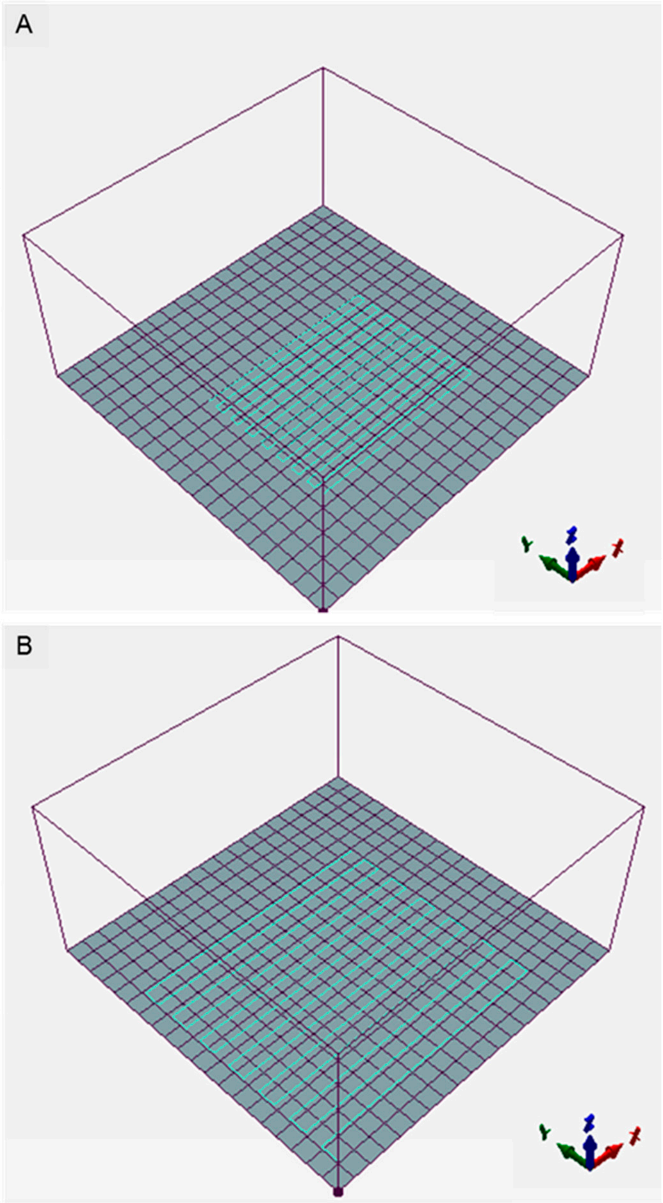

Table S1. List of microorganisms and type of viability test used.

Microbial species	Functional type	Viability test
Trichoderma harzianum	Fungus that belong to the Ascomycota division. Mycoparasitic and capable of inducing resistance in plants, it is widely used as a biocontrol agent.	Spore germination and spore production over agarized, solid substrate
Coniothyrium minitans	Fungus that belong to the Ascomycota division. Mycoparasitic used as a biocontrol agent.	Spore germination and spore production over agarized, solid substrate
Glomus mosseae	Fungus that belong to the Glomeromycota division. Capable of mycorrhizal interaction and used as a biostimulant and biocontrol agent.	Spore germination
Bacillus subtilis	Gram-positive bacterium belonging to the phylum Firmicutes. Widely used as a biocontrol agent.	Growth in liquid substrate
Pseudomonas fluorescens	Gram-negative bacterium belonging to the phylum Proteobacteria. Widely used as a biocontrol agent.	Growth in liquid substrate

Table S2. List of herbicides, assay plants and concentrations used. The concentrations are reported in µl ml^{-1}, differently from what is reported on the label for most plant protection products (l ha^{-1}).

Active compound and commercial name	Plant species	Concentration
Glifosate (Clinic 360)	*Lolium temulentum*	30 µl ml^{-1}<br>10 µl ml^{-1}<br>3 µl ml^{-1}<br>1 µl ml^{-1}<br>0.3 µl ml^{-1}
Glifosate (Clinic 360)	*Avena fatua*	30 µl ml^{-1}<br>10 µl ml^{-1}<br>3 µl ml^{-1}<br>1 µl ml^{-1}<br>0.3 µl ml^{-1}
Cicloxidim (Stratos)	*Lolium temulentum*	10 µl ml^{-1}<br>3 µl ml^{-1}<br>1 µl ml^{-1}<br>0.3 µl ml^{-1}<br>0.1 µl ml^{-1}
Cicloxidim (Stratos)	*Avena fatua*	10 µl ml^{-1}<br>30 µl ml^{-1}<br>1 µl ml^{-1}<br>0.3 µl ml^{-1}<br>0.1 µl ml^{-1}

Table S3. List of insecticides, species of insects tested, and concentrations used. The concentrations are reported in µl ml^{-1}, differently from what is reported on the label for most plant protection products (l ha^{-1}).

Active compound and commercial name	Insect species	Concentration
Abamectine (VERTIMEC)	*Galleria mellonella*	1 µl ml^{-1}<br>0.3 µl ml^{-1}<br>0.1 µl ml^{-1}<br>0.03 µl ml^{-1}<br>0.01 µl ml^{-1}
Abamectine (VERTIMEC)	*Sarcophaga carnaria*	1 µl ml^{-1}<br>0.3 µl ml^{-1}<br>0.1 µl ml^{-1}<br>0.03 µl ml^{-1}<br>0.01 µl ml^{-1}
Deltametrine (DECIS)	*Galleria mellonella*	0.6 µl ml^{-1}<br>0.1 µl ml^{-1}<br>0.06 µl ml^{-1}<br>0.01 µl ml^{-1}<br>0.006 µl ml^{-1}
Deltametrine (DECIS)	*Sarcophaga carnaria*	0.6 µl ml^{-1}<br>0.1 µl ml^{-1}<br>0.06 µl ml^{-1}<br>0.01 µl ml^{-1}<br>0.006 µl ml^{-1}

Table S4. List of beneficial microbes, plants assays and concentrations used. Concentrations are reported as colony forming units (CFU) per mL of product.

Beneficial microbe and commercial name	Biological activity	Plant species	Concentration
Trichoderma harzianum (SAMAGRI)	Biostumulant, mycoparasite, induce plant resistance	*Lolium perenne*	$1x10^7$ ml^{-1}<br>$1x10^6$ ml^{-1}<br>$1x10^5$ ml^{-1}<br>$1x10^4$ ml^{-1}<br>$1x10^3$ ml^{-1}
Trichoderma harzianum (SAMAGRI)	Biostumulant, mycoparasite, induce plant resistance	*Eruca vesicaria*	$1x10^7$ ml^{-1}<br>$1x10^6$ ml^{-1}<br>$1x10^5$ ml^{-1}<br>$1x10^4$ ml^{-1}<br>$1x10^3$ ml^{-1}
Bacillus subtilis (SERENADE)	Biostumulant and biocontrol	*Lolium perenne*	$1x10^7$ ml^{-1}<br>$1x10^6$ ml^{-1}<br>$1x10^5$ ml^{-1}<br>$1x10^4$ ml^{-1}<br>$1x10^3$ ml^{-1}
Bacillus subtilis (SERENADE)	Biostumulant and biocontrol	*Eruca vesicaria*	$1x10^7$ ml^{-1}<br>$1x10^6$ ml^{-1}<br>$1x10^5$ ml^{-1}<br>$1x10^4$ ml^{-1}<br>$1x10^3$ ml^{-1}

Table S5. List of treatments and variables considered in the first experiment with hydrosensitive papers sheet.

Sprayer system	Liquid	Height of application
ST20	Water	10 cm
ST50	Water	10 cm
Spray	Water	10 cm

Table S6. List of variables included in the experiment with beneficial microorganisms on microgreens. The concentrations of the fungus *Trichoderma harzianum* are reported as colony forming units (CFU) per ml of commercial product.

Beneficial microbe and commercial name	Sprayer system	Height of application	Plant species	Concentration
Trichoderma harzianum (SAMAGRI)	ST20	10, 15, 20 cm	*Eruca vesicaria* *Lactuca sativa*	$1x10^5$ ml^{-1}
Trichoderma harzianum (SAMAGRI)	ST50	10, 15, 20 cm	*Eruca vesicaria* *Lactuca sativa*	$1x10^5$ ml^{-1}
Trichoderma harzianum (SAMAGRI)	Spray	10, 15, 20 cm	*Eruca vesicaria* *Lactuca sativa*	$1x10^5$ ml^{-1}
